# Supplementary material for: Autophagy Dynamics and Modulation in a Rat Model of Renal Ischemia-Reperfusion Injury
Source: Int J Mol Sci. 2020 Sep 29;21(19):7185. doi: 10.3390/ijms21197185 (PMC7583807; doi:10.3390/ijms21197185)
Supplement: Supplementary file 1 [file ijms-21-07185-s001.zip › Supplemental Figure Legends.pdf]

Supplemental Figure Legends

Fig. S1: Kidney injury is increased and tissue inflammatory processes are activated after mild ischemia and reperfusion. Rats, either Sham-operated (Sham) or subjected to 45 min of renal ischemia (I45), were sacrificed at various time points post-reperfusion (R0h, R1h, R3h, R6h, R24h, R48h, R7d and R90d). Kidney sections were stained with TUNEL (A) or analyzed with transmission electron microscopy (B), revealing mitochondria (arrowheads), early autophagosomes (a) and late autophagosomes (*). Kidney tissue was analyzed for mRNA expression of IL-10 (C) and IL-6 (D). * $p < 0.05$, ** $p < 0.01$.

Fig. S2: Autophagy is suppressed post-reperfusion following mild ischemia. Rats, either Sham-operated (Sham) or subjected to 45 min of renal ischemia (I45), were sacrificed at various time points post-reperfusion (R0h, R1h, R3h, R6h, R24h, R48h, R7d and R90d). Kidneys were collected and analyzed by Western blotting for the annotated markers.

Fig. S3: Apoptosis increases post-reperfusion following mild ischemia. Rats, either Sham-operated (Sham) or subjected to 45 min of renal ischemia (I45), were sacrificed at various time points post-reperfusion (R0h, R1h, R3h, R6h, R24h, R48h, R7d and R90d). Kidney tissue was collected and analyzed by Western blotting for the annotated markers.

Fig. S4: Expression of IL-6 and IL-10 is reduced following severe ischemia. Rats, either Sham-operated (Sham) or subjected to 45 min (I45) or 60 min (I60) of renal ischemia, were sacrificed at various time points post-reperfusion (R0h, R3h and R24h). Kidney tissue was collected and analyzed for mRNA expression of IL-6 (A) and IL-10 (B). * $p < 0.05$, ** $p < 0.01$, *** $p < 0.001$.

Fig. S5: Severe ischemia and reperfusion after severe ischemia does not affect apoptosis. Rats, either Sham-operated (Sham) or subjected to 45 min (I45) or 60 min (I60) of renal ischemia, were sacrificed at various time points post-reperfusion (R0h, R3h and R24h). Kidneys were collected and analyzed by Western blotting for the annotated markers (A-C) and quantified for Bax (D), Bcl-2 (E) and cleaved Caspase 3 (F). Kidney tissue was analyzed by qPCR for mRNA expression of Bax (G), Bcl-2 (H) and Bim (I).The relative change of I45 and I60 compared to the corresponding Sham group (represented by the dashed line at y=1) was plotted. * $p < 0.05$, ** $p < 0.01$.

Fig. S6: Trehalose does not alter mRNA expression of inflammatory and injury markers. Rats were injected with vehicle (Veh) or trehalose 48h and 24h prior to 60 min of renal ischemia (I60), and sacrificed 24h post-reperfusion. Kidneys were analyzed by qPCR for mRNA expression of ICAM-1 (A), Hsp70 (B), IL-6 (C) and IL-10 (D).
