# Supplementary figures and images for: Autophagy Dynamics and Modulation in a Rat Model of Renal Ischemia-Reperfusion Injury
Source: Int J Mol Sci. 2020 Sep 29;21(19):7185. doi: 10.3390/ijms21197185 (PMC7583807; doi:10.3390/ijms21197185)

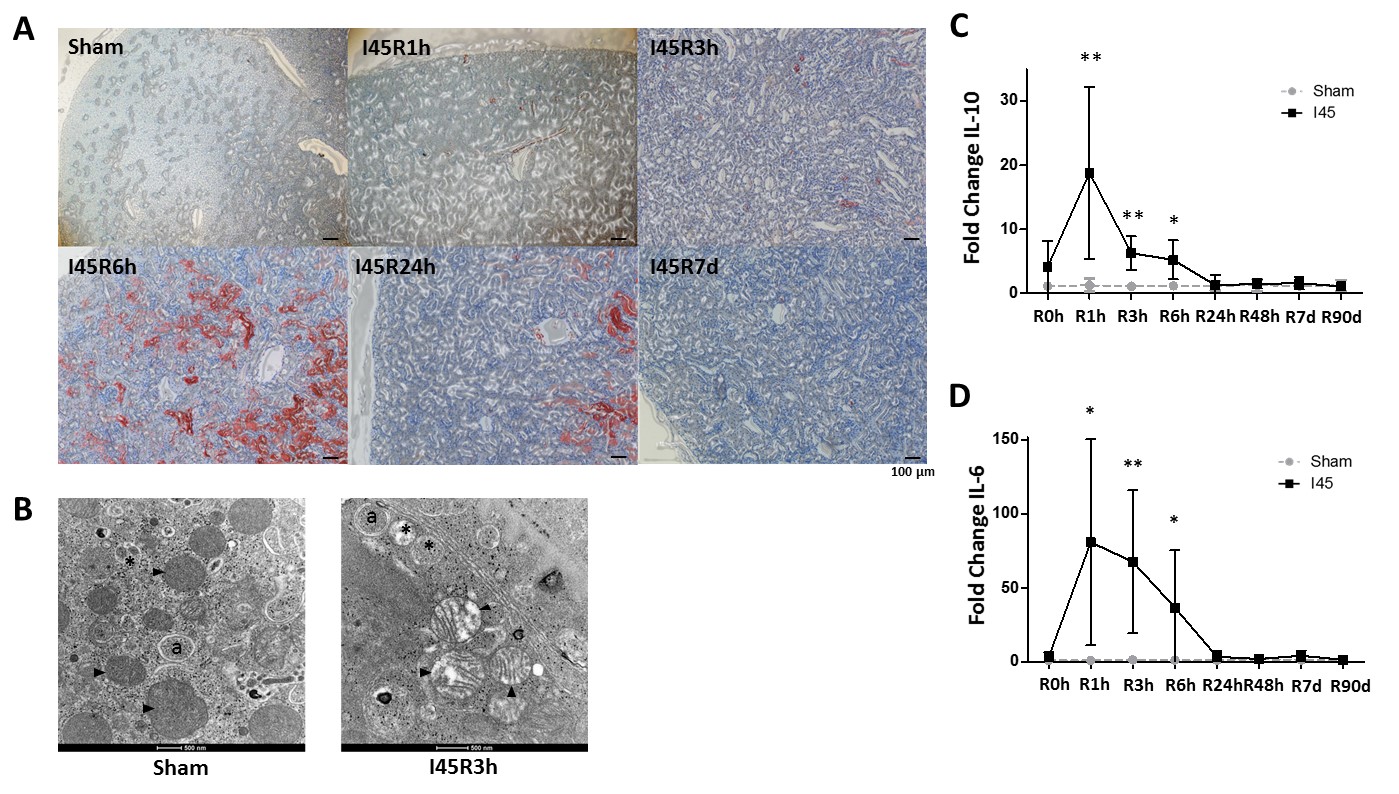

Supplement: Supplementary file 1 [file ijms-21-07185-s001.zip › Fig. S1.jpg]

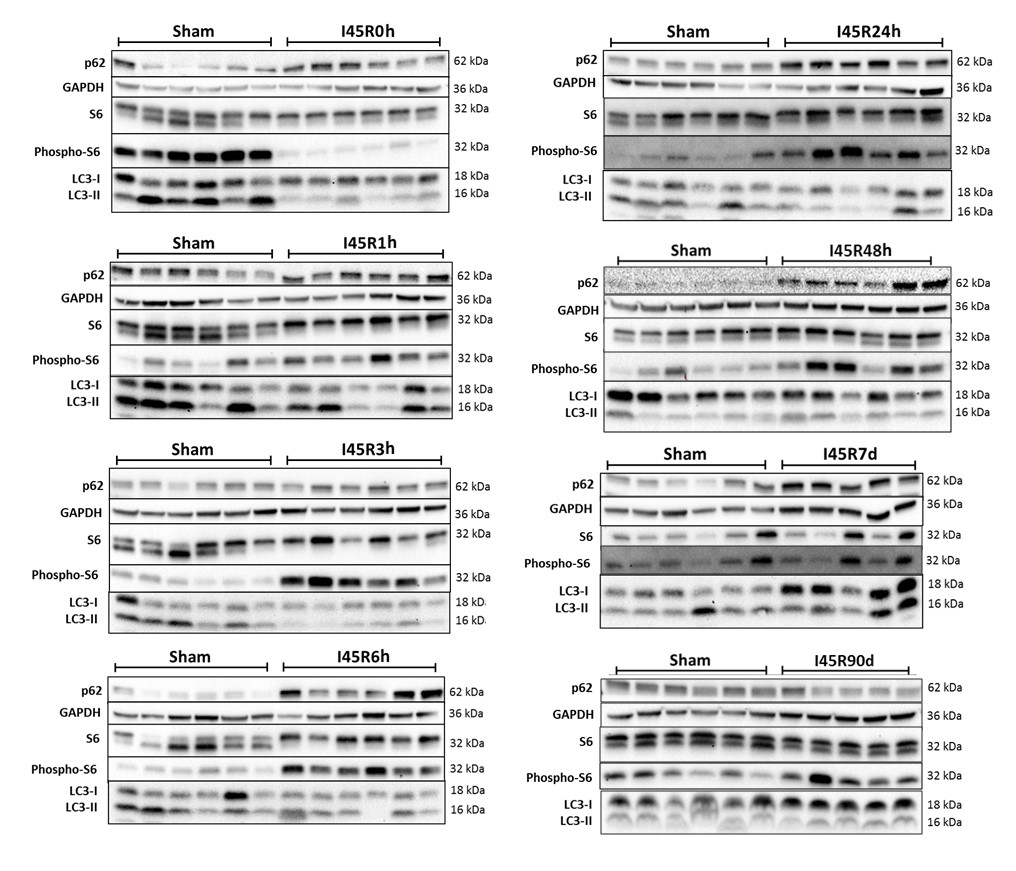

Supplement: Supplementary file 1 [file ijms-21-07185-s001.zip › Fig. S2.jpg]

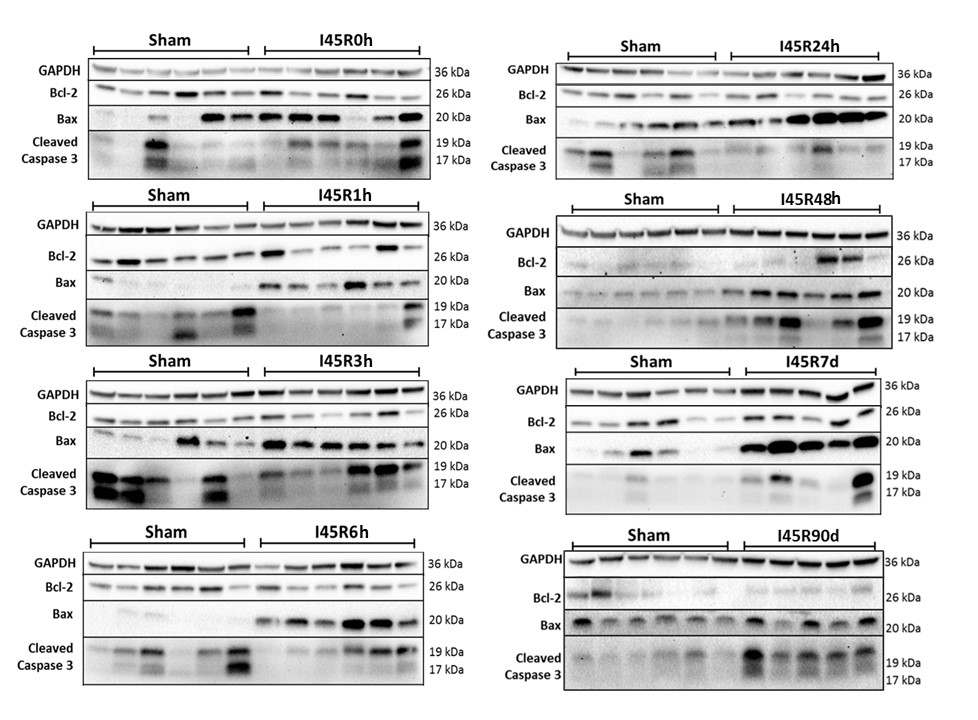

Supplement: Supplementary file 1 [file ijms-21-07185-s001.zip › Fig. S3.jpg]

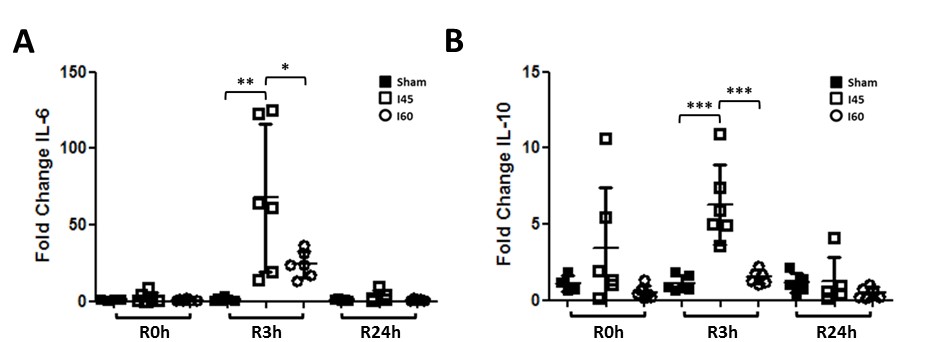

Supplement: Supplementary file 1 [file ijms-21-07185-s001.zip › Fig. S4.jpg]

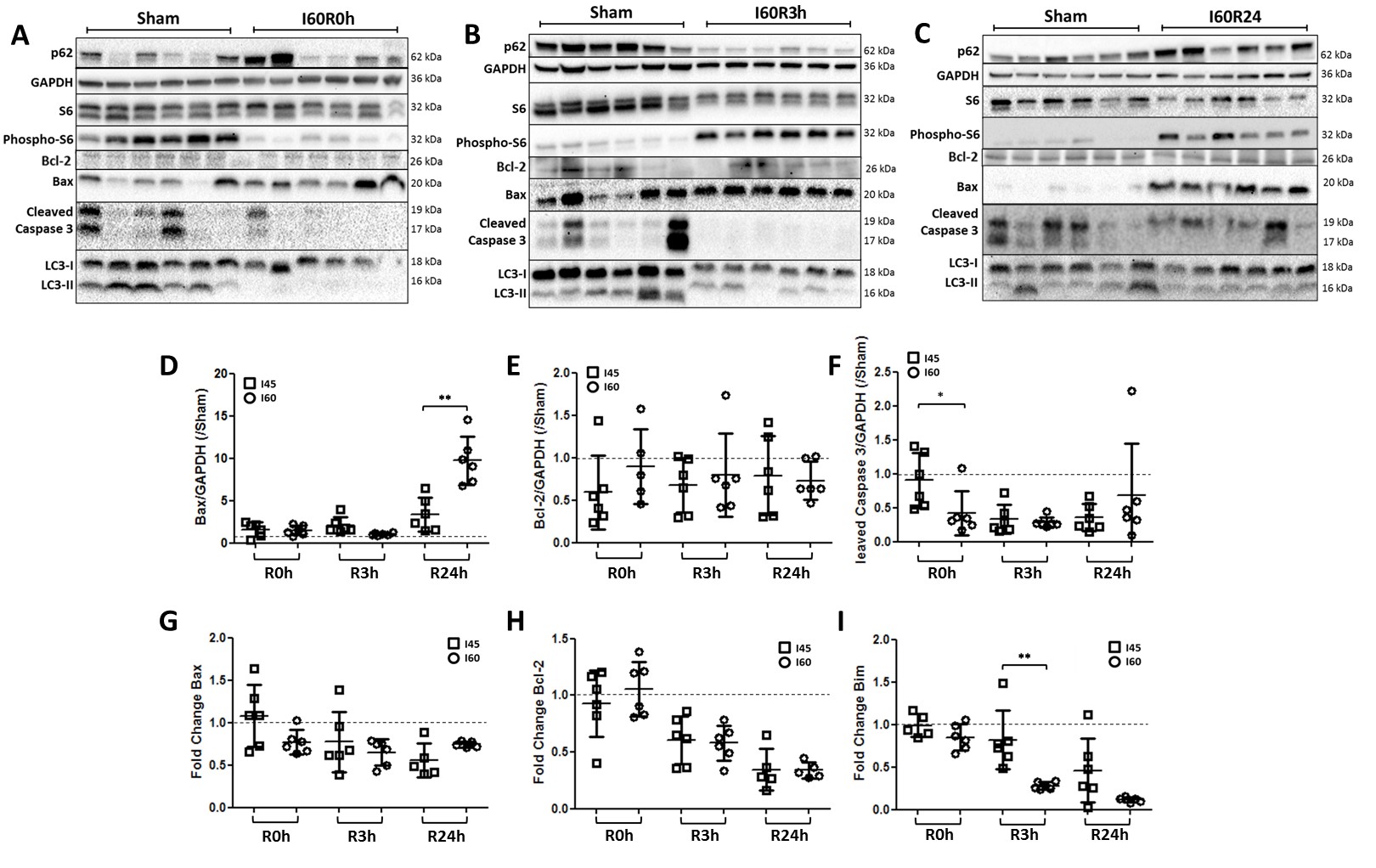

Supplement: Supplementary file 1 [file ijms-21-07185-s001.zip › Fig. S5.jpg]

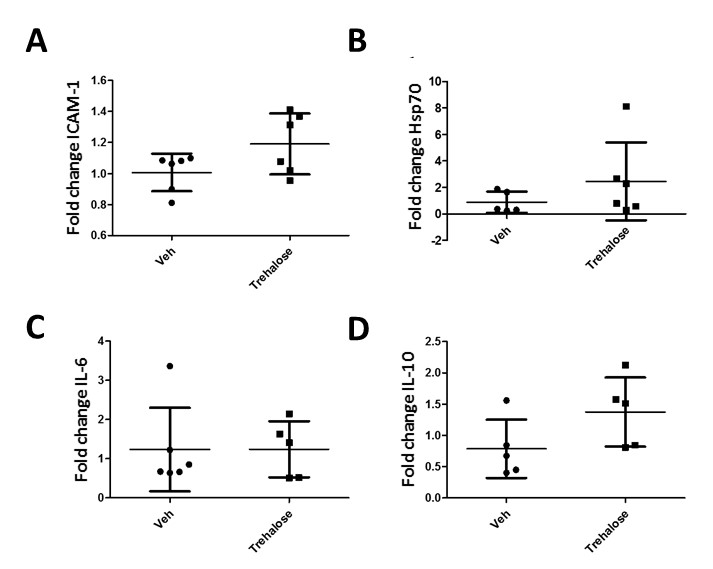

Supplement: Supplementary file 1 [file ijms-21-07185-s001.zip › Fig. S6.jpg]
